# Enhancing workplace learning at the transition into practice: Lessons from a pandemic

**DOI:** 10.1111/medu.14240

**Published:** 2020-06-30

**Authors:** Hannah Gillespie, Florence Findlay White, Neil Kennedy, Tim Dornan

## 1 WHAT PROBLEMS WERE ADDRESSED?

Taking responsibility for prescribing is one of newly qualified doctors' greatest stressors.^1^ Despite being a routine task, prescribing insulin is particularly stress‐inducing. The coronavirus disease 2019 (COVID‐19) pandemic has made it more important to minimise the stress experienced by transitioning students, yet there are fewer clinicians to support their accelerated transitions.

We had planned an intervention during the 9‐week ‘clinical assistantships’ that students undertake immediately before he or she qualifies. Students were to write insulin ‘pre‐prescriptions,’ which supervisors would endorse as prescriptions that were appropriate to dispense. Trained health care professionals or individuals with diabetes (debriefers) were to conduct one‐to‐one case‐based discussions (CBDs) to help students learn reflectively from his or her experience.

We were unable to carry this out as intended: clinical placements were cut short and students who had been exposed to COVID‐19 posed a risk to our debriefers with diabetes. Despite that, we set out to enhance, rather than abandon, our novel form of reflective, experiential education.

## 2 WHAT WAS TRIED?

We encouraged students to gain experience of pre‐prescribing insulin for hospitalised patients, as intended, but under the COVID‐19 pandemic conditions. Face to face CBDs were impossible, yet one‐to‐one reflective discussions play an essential part in students’ learning and therefore we made a rapid decision to migrate these sometimes very personal discussions on to an online videoconferencing platform Zoom™ (Zoom Video Communications Inc., San Jose, CA, USA).

A curriculum administrator took over responsibility for booking CBDs from 11 teaching hospitals. The central organisation of technology‐supported reflective conversations made optimum use of available resources, aligned the availabilities of debriefers and students, minimised missed appointments and made a CBD available to every interested student.

We revised our operating procedures so that, as intended, deep reflective discussions took place, allowing students to reflect on experiences and make commitments to safe and appropriate future prescribing behaviour. Records of these CBDs provided rich, anonymised descriptions of students’ reflective learning; qualitative analysis of these allowed us to evaluate the intervention.

## 3 WHAT LESSONS WERE LEARNED?

A total of 77 students (approximately 25% of the cohort) participated. Detailed records were available for 25 of these students, who both wrote an insulin pre‐prescription and completed a voluntary CBD, yielding over 7000 words of evaluation data for qualitative analysis.

The COVID‐19 pandemic helped students learn three key lessons: (a) participants who had navigated indeterminate situations, in which factors such as COVID‐19 created ambiguity and made prescribing decisions complex, were able to verbalise how he or she would manage experiences like these in future; (b) reflecting on an instance of prescribing helped students situate individual prescribing decisions in the wider context of patients’ trajectories of care, in which insulin prescribing must be reconciled with other medications and co‐morbidities, and (c) participants learned he or she were not alone and could develop collaborative expertise by seeking help from fellow professionals, patients and senior colleagues.

We learned lessons too: (a) it is tempting to abandon workplace education when students are numerous and clinicians are busy, yet the COVID‐19 pandemic conditions encouraged us to use good learning opportunities creatively; (b) transitioning to an online communication platform made supporting student reflection on workplace learning so much easier that conducting CBDs online is our new normal, and (c) helping students seek out educational opportunities can ‘engage their brains’ and encourage them to ‘ask why things are being done the way they are.’ This applies to medical education as much as it does to clinical medicine.
